# Rheology and Processing of Polymers

**DOI:** 10.3390/polym14122327

**Published:** 2022-06-08

**Authors:** Khalid Lamnawar, Abderrahim Maazouz

**Affiliations:** 1 CNRS, UMR 5223, Ingénierie des Matériaux Polymères, INSA Lyon, Université de Lyon, F-69621 Villeurbanne, France; 2 University Jean Monnet, F-42100 Saint-Étienne, France; 3 Hassan II Academy of Science and Technology, Rabat 10100, Morocco; abderrahim.maazouz@insa-lyon.fr

I am so glad to share with you our Special Issue entitled ‘Rheology and Processing of Polymers’, which covers the latest developments in the field of rheology and polymer processing, highlighting cutting-edge research focusing on the processing of advanced polymers and their composites. It demonstrates that the field of “Rheology and Polymer Processing” is still gaining attention.

This Special Issue promises to include a variety of papers to serve both academia and industry communities alike. After a meticulous review and editing process, we are delighted to have finally accepted 15 papers and 1 review. We thank the authors for their contributions and the peer reviewers for their thorough and thoughtful reviewing.

In sum, the published review and papers cover a wide range of topics, from polymer science to recycling, recyclability and the reuse of polymers and their multiphase systems.

The following keywords cover all related topics:

- polymer processing;
- rheology;
- polymers;
- natural polymers and biopolymers;
- biopolymers;
- polymer nanocomposites;
- advanced polymers;
- composites and biocomposites;
- biocomposites;
- modeling;
- numerical simulation;
- polymer physics;
- innovative processing;
- polymer melts;
- polymer engineering;
- recycling

Bo Lu et al. [1] reviewed interfacial phenomena in multi-micro/nanolayered polymer coextrusion from fundamental and engineering aspects. Different from traditional approaches, such as layer-by-layer assembly and spin coating which suffer from low productivity, multilayer coextrusion processing is a top-down approach able to industrially manufacture multilayered films. These phenomena, during processing, including interlayer diffusion, interlayer reaction, interfacial instabilities and interfacial geometrical confinement, frequently occur at the interfaces of multilayered polymers in multilayer coextrusion processing. For example, the spatial confinement of layers greatly alters the microstructure and dynamics of multilayer polymers, dramatically influencing macroscopic properties, including mechanical, electric and gas/liquid barrier properties. This review clearly illustrates the origin of these interfacial phenomena and how they can affect the microstructural development and resulting macroscopic properties. In the future, more comprehensive work should be carried out in both academia and the industry to further clarify these interfacial phenomena toward the scaling up of multilayer coextrusion.

Najoua Barhoumi et al. [2] designed a sort of fluorinated ethylene propylene (FEP) polymer coating with the aim of protecting the stainless-steel (SS304) mold substrate surface from corrosion and wear. Through an air spray process and curing, a compact and uniform film was obtained, and the mechanical, adhesion and corrosion, etc., properties were tested. The FEP coating exhibited a dense, poreless and homogeneous structure with a pseudohexagonal lattice crystalline structure. It showed good mechanical properties, hardness, Young’s modulus and good scratch resistance to adhesive and cohesive failure, with a high adhesion to SS304. The multipass scratch test provided an easy and quick approach to study the FEP coating’s wear resistance. The friction coefficient of the FEP coating did not exceed 0.13, and the wear coefficient was approximately 3.12 × 10^−4^ mm^3^ N m^−1^. The FEP coating enhanced the corrosion resistance of SS304 and provided significant protection during a 60-day immersion in a NaCl solution.

Raffael Rathner et al. [3] investigated the suitability of the Cox–Merz rule to linear high-density polyethylene (HDPE) materials with various molecular masses. The Cox–Merz rule is an empirical relationship that is commonly used in science and the industry to determine shear viscosity on the basis of an oscillatory rheometry test. However, it does not apply to all polymer melts. Three rheological measurement techniques were used to determine the shear-rate-dependent viscosity of three different HDPE materials: an oscillatory parallel plate, high-pressure capillary and extrusion slit rheometry. While in parallel plate rheometry the Cox–Merz relation was used to estimate the shear-dependent viscosity, in high-pressure capillary and extrusion slit rheometry, the Weissenberg–Rabinowitsch relation was employed. The results showed that for the HDPE grades tested, the viscosity data based on the capillary pressure flow of the high-molecular-weight HDPE described the pressure drop inside the pipe head significantly better than data based on parallel plate rheometry applying the Cox–Merz rule. For the low-molecular-weight HDPE, both measurement techniques were in good accordance.

Linear and branched polybutylene succinate (PBS) blends were created by Violette Bourg et al. [4] to investigate their molecular architecture and rheological behavior. Long-chain branched (LCB) structures have already demonstrated their efficiency in improving the melt strength compared to the linear structures. LCB polymers in general, and in particular newly developed (bio)polymers, are more expensive than linear polymers due to the more complex synthesis or additional reactive extrusion step to obtain them. Blending linear and branched PBS is, therefore, an interesting concept in regards to improving the processability of linear polymers, while decreasing their price. According to their results, when the branched PBS (BPBS) weight fraction increased, a clear increase in the weighted relaxation strengths was observed. Meanwhile, under elongational flow, the rheological response of blends changed from shear thinning to strain hardening with the increase in strain rate. This study provided a deep structural and rheological characterization of the long-chain branching and linear PBS, helping to understand the influence of their blending on linear and nonlinear viscoelastic properties.

Angelica Avella et al. [5] compared crosslinking efficiency between a water-assisted reactive melt processing (REx) and a slower PCL macroradicals diffusion in the melt state. The results showed that the presence of water increased the PCL molecular weight and gel content compared to the dry process, from 1% to 34% with 1 wt.% peroxide, confirming a more efficient radical propagation in water-assisted REx. After Rex, a differential scanning calorimetry showed increased crystallization temperatures and an easier crystallization process of reacted PCL, compared to neat PCL. From the dynamic mechanical analysis, higher branching/crosslinking slightly increased the transition temperatures and led to a reinforcement effect in the temperature range below the glass transition. After the glass transition, the mechanical reinforcement was limited. Instead, in the melt state, the effect of branching/crosslinking was more evident as shown by melt rheology. The rheological behavior of crosslinked PCL showed a transition from the typical viscous-like to a solid-like state. Shear storage moduli were increased by the reactive melt processing, confirming the desired improvement of PCL rheological properties. This work represents a relevant reference for the future controlled water-assisted reactive melt processing of polymers and composites, in which water could have the role of feeding media, e.g., for polysaccharides suspension, but also be useful for boosting peroxide-initiated reactions.

Geraldine Cabrera et al. [6] developed a novel approach for commonly found complex polyethylene/polypropylene (PE/PP) and polyethylene/polystyrene (PE/PS) systems in industrial waste streams from design to recycling. The method involves moving from eco-design to the mechanical recycling of multilayer films via forced assembly coextrusion. PE/PP and PE/PS multilayer films were prepared by coextrusion with various numbers of layers, and then recycled using a process including steps such as (i) grinding, (ii) monolayer cast film extrusion or (iii) injection molding, with or without an intermediate blending step through twin-screw extrusion. For the PE/PP system, the mechanical strength of the multilayer films was apparently enhanced when the most suitable configuration was chosen. For the PE/PS system, the multilayered films presented a brittle behavior with a nominal thickness at the microscale. However, as the nominal layer thickness was decreased down to the nanoscale, the multilayer films became more ductile as the elongation at the break increased. Regardless of the polymer system studied, it was demonstrated that the design of multi-micro/nanolayer films is a very promising solution for industrial issues that accompany the valorization of recycled materials, without the use of compatibilizers.

Because of the lack of commercial filaments for conventional fused deposition modeling (FDM) method, the three-dimensional (3D) printing of polycaprolactone/ nanohydroxylapatite (PCL/nHA) nanocomposites seems to be very tough work, whereas Pang-Yun Chou et al. [7] fabricated PCL/nHA nanocomposites using a lab-developed solution-extrusion printer. The effects of distinct printing variables on the mechanical properties of nanocomposites were investigated. The tensile property of printed nanocomposites increased with the fill density, yet diminished with a decrease in the ratio of PCL/nHA to DCM and print speed. Nanocomposite parts printed with a 90° orientation demonstrated the most superior mechanical properties. In addition, the drug-loaded PCL/nHA screws were also prepared via the same technique, which could provide an extended elution of high levels of vancomycin/ceftazidime over a 14-day period. As a result, this kind of solution-extrusion 3D printing technology may be used to print drug-loaded implants for various medical applications.

Geunyeop Park et al. [8] explained the nature of stability curves in the spinning process of Giesekus fluids—the initial stabilization and subsequent destabilization pattern with respect to D_e_ in the intermediate range of the material parameter. Various theoretical approaches were considered in this study to examine the changes in the stability curves with respect to the material parameter of Giesekus fluids in the isothermal spinning process without cooling, including steady velocity profiles and extensional deformation properties in the spinline, and kinematic waves traveling along the entire spinline. The material parameter α_G_ in this fluid model suitably depicted the extensional thickening (stabilizing effect of D_e_) and extensional thinning (destabilizing effect of D_e_) properties of viscoelastic fluids in extensional deformation processes. In the intermediate range of values of α_G_ (approximately 0.01 < α_G_ < 0.4), the effect of D_e_ on the stability was unusual—the system was stabilized in the low and medium D_e_ regions and then destabilized in the high D_e_ region. This tendency may be qualitatively interpreted by extensional flow characteristics in the spinline. When D_e_ increased at a fixed drawdown ratio (e.g., r = 25 condition applied in this study) in the intermediate α_G_ region, the system, starting from the unstable state became stable after the first onset for low or medium D_e_, resulting in a higher level of spinline velocity and strain-hardening viscosity. It became unstable again beyond the second onset for high D_e_, yielding a lowered spinline velocity and an insignificant strain-hardening feature. A combination of transit times of the kinematic waves penetrating the entire spinline and period of oscillation, i.e., the simple indicator from the linear stability analysis, predicted well the draw resonance onsets under different D_e_ and α_G_ conditions. It was confirmed that these transit times of kinematic waves for varying D_e_ adequately reflected the dependence of change in stability on the values of α_G_.

Francisco Parres et al. [9] studied the influence of wood flour particle size, at the microscale level, on the properties of biodegradable composite materials based on Solanyl^®^-type bioplastic. Wood flour with different granulometries, ranging from 70 to 1100 µm, was assessed as a filler. The rheological study revealed an increase in viscosity as the filler percentage increased. Despite the low viscosity variation as the percentage of filler increased, a more detailed analysis showed that pressures rose more quickly, which could be attributed to the obstruction of the nozzle due to the presence of wood flour particles. This fact could increase the number of defective pieces due to the obstruction of the gate inside the mold. All these results indicated that the material could be processed by extrusion. The tensile test results showed that, in general, Young’s modulus provided increasing values for all types of particles. Similarly, the tensile strength increased with an increasing amount of wood flour small-sized (CB 120) and medium-sized particles (BK 40–90) in the formulation. The elongation at break showed little variation with the addition of different percentages of medium-sized particles (BK 40–90) and the largest particles (Grade 9) to the Solanyl^®^-type bioplastic. Nevertheless, the incorporation of the smallest lignocellulosic microparticles (CB 120) increased the elongation at the break. The impact strength was only affected by the incorporation of small- and medium-sized particles (CB 120 and BK 40–90), showing a maximum value for biocomposites with a 20 wt.% wood flour filler. Of all the samples analyzed, those with a 10 or 20 wt.% filler of the smallest particles (CB120) and medium-sized particles (BK 40–90) showed the best combination for processing by injection molding, as well as leading to biocomposites with improved properties and, thus, ones of interest for sustainable industrial applications.

Fouad Erchiqui et al. [10] assessed the reliability of the experimental method for the viscoelastic identification of a nanocomposite reinforced with polymethylsilsesquioxane nanoparticles (PMSQ–HDPE). Two tests of different nature were used, one based on the free inflation of the membrane and the other on a dynamic mechanical test (DMA). The experiments were carried out at a temperature of 130 °C. The material constants for Christensen’s model were determined by the least square optimization. The comparative study of the viscoelastic behavior of PMSQ–HDPE showed that the biaxial test was more appropriate for the construction of a behavior law for applications in thermoforming. Concerning the viscoelastic identification obtained from the rheological data of the DMA, it did not seem to be capable of representing the thermoforming of a part, which requires large deformations. Following this study, comparative studies between the DMA and free blowing should be carried out at temperatures above 130 °C for viscoelastic identification, making it possible to characterize the effect of temperature on the reliability of the tests in thermoforming.

Jie Wang et al. [11] investigated the multiscale structural development involving both the layer architecture and microstructure within layers of micro/nanolayer coextruded polymer films, as well as their relationship to dielectric properties based on the poly (vinylidene fluoride-co-hexafluoropropylene) (PVDF-HFP)/polycarbonate (PC) system. The layers were stable and continuous for microlayer coextruded films with nominal layer thicknesses, whereas for nanolayer coextruded films at nominal layer thicknesses below 160 nm, layer integrity was reduced by interfacial instabilities triggered by viscoelastic differences between component melts. Layers even broke into microdroplets and microsheets due to the coalescence of thin layers. Moreover, with the reduction in the nominal layer thickness, the films displayed an enhanced crystallization, with the formation of a 2D oriented spherulite structure in microlayer coextruded systems and highly oriented in-plane lamellae in nanolayer coextruded counterparts. Layer breakup in thinner layers further resulted in a crystallization and structural orientation similar to that found in microlayer films, which was attributed to the relaxation and phase coalescence of thin layers during processing. Furthermore, dielectric properties of films were strongly dependent on these multiscale structures. The gradually increasing storage permittivity upon reducing the layer thickness was ascribed to the enhanced molecular orientations that could facilitate dipole switching. The lower storage permittivity in the thin layers with breakup was caused by the reduced orientations. In addition, the dielectric loss of microlayer coextruded films was lower than that of nanolayer coextruded analogues. This was due to the increased hopping of charge carriers and the higher energy dissipation from dipole switching when layers were broken. The results of this study could allow for a better understanding of the multiscale structure evolution in micro/nanolayer coextrusion to optimize the target macroscopic properties.

Mei Fang et al. [12] studied the aging process of carbon fiber (CF)/polycarbonate (PC) composites under a humid and hot environment. The moisture absorption rate was measured and calculated during the aging process. Changes in mechanical properties before and after hydrothermal aging were measured through tensile and flexural tests. Underlying mechanisms for hydrothermal aging-induced changes in mechanical and sand erosion properties were surveyed. The moisture absorption rate of CF/PC composites grew linearly with the square root of aging time during the first five days, and stayed flat upon reaching saturation. On the premise of a sampling period and frequency every seven days, the tensile properties reached their maximum at the seventh day, and the peak value of bending flexural performance was reached on the fourteenth day. The effects of aging time on the storage modulus and solid particle erosion resistance were consistent with the stretching results. Hydrothermal aging caused holes to form on the surface of CF/PC composites and reduced the specimen’s sand erosion resistance. Meanwhile, the physical property of CF/PC composites was changed.

Cindy Le Hel et al. [13] studied the evolution of morphology at a high-level crosslinked elastomer phase in a thermoplastic matrix, and explained how such a level of concentration could be reached. Meanwhile, the influence of the concentration of the elastomer (EPDM) phase on the recovery elasticity of these thermoplastic vulcanizates (TPVs) as well as the influence of reinforcing fillers such as carbon black were also investigated. The phase inversion between both phases took place when the EPDM phase reached a certain level of crosslinking (swelling ratio ~20 corresponding to a crosslinking density of 10 mol/m^−3^), which allowed the stress applied by the thermoplastic phase (PP) matrix to disperse this phase by fragmentation. Maximal stress was observed at the melting temperature of the PP pellets. The crosslinking chemistry nature (radical initiator or phenolic resin crosslinker) had less influence on the elasticity recovery in the case of the TPV compared with a pure EPDM system. Morphology was the first-order parameter, whereas the crosslinking density of the EPDM phase was a second-order parameter. The morphologies of the peroxide-based TPV showed heterogeneous systems with large EPDM domains. However, this large distribution of EPDM particles is the only way to increase the maximal packing fraction of the crosslinked EPDM phase, which behave as solid particles. The addition of carbon black fillers allowed the mechanical properties at the break to significantly improve at the lowest PP concentration (20%). This improvement of mechanical properties is still not well understood. Finally, to reach the best compression set while keeping good mechanical properties, a peroxide-based TPV containing carbon black and as little PP as possible should be formulated. Therefore, the low quantity of PP allows for a good compression set; the peroxide permits the carbon black to be located at the interface between the two phases and, thus, the filler can reinforce the mechanical properties of the material.

To effectively improve the processability and toughness of magnesium hydroxide (MH)/linear low-density polyethylene (LLDPE) composites, a polysiloxane grafted with a thermotropic liquid crystal polymer (PSCTLCP) was designed and synthesized by Xiaoxiao Guan et al. [14]. The effect of PSCTLCP loading on the processability and tensile properties of MH/LLDPE/PSCTLCP composites was studied. Moreover, the modified mechanism of PSCTLCP on MH/LLDPE/PSCTLCP composites was investigated at different processing temperatures, rotation speeds and shear rates to guide the actual processing in industry. The balance melt torque of MH/LLDPE/PSCTLCP composites obviously decreased with the loading of PSCTLCP, and that of the composite (5.0 wt.% loading) decreased from 16.59 N·m of the baseline MH/LLDPE to 9.67 N·m. Moreover, PSCTLCP weakened the non-Newtonian property and decreased the flowing activation energy of MH/LLDPE/PSCTLCP composites, and, thus, broadened the processing window and improved the processability. When considering the flexible polysiloxane as the main chains of PSCTLCP, the elongation at the break of MH/LLDPE composites significantly increased, and the tensile strength and modulus slightly decreased with PSCTLCP loading.

The long-chain branching (LCB) of polypropylene (PP) is regarded as a game changer in foaming due to the introduction of strain hardening, which stabilizes the foam morphology. A thorough characterization with respect to the rheology and crystallization characteristics of a linear PP Sinopec HMS20Z, a PP/PE-block copolymer Sinopec E02ES CoPo and a long-chain branched PP Borealis WB140 HMS was conducted by Nick Weingart et al. [15]. Although only LCB-PP exhibited strain hardening and had five times the melt strength of the other grades, it did not provide the broadest foaming window or the best foam quality in terms of density (140 g/L) and cellular morphology. On the contrary, the linear Sinopec HMS20Z delivered low densities (<40 g/L) and superior foam morphology. The Sinopec E02ES CoPo performed rather negatively in terms of density and mediocre in terms of cellular morphology. The beneficial foaming behavior of the Sinopec HMS20Z-PP was attributed to a slower crystallization and a low crystallization temperature compared to the other two materials. Multiwave experiments were conducted to study the gelation due to crystallization. In isothermal multiwave experiments, the Sinopec HMS20Z exhibited a gel-like behavior over a broad time frame, whereas the other two PPs froze quickly. Nonisothermal multiwave tests also underlined the finding of a broad processing window for the linear PP (Sinopec HMS20Z) as the temperature (and time) difference between the onset of crystallization and the gel point was significantly broader compared to the other PP grades. These findings were also confirmed in DSC experiments, where crystal perfection occurred notably slower for Sinopec HMS20Z, which in turn led to a longer gel-like state before solidification. Again, it was noted that this PP grade exhibited no strain hardening. Thus, it was concluded that, besides sufficient rheological properties, the crystallization behavior is of utmost importance for foaming in terms of a broad processing window, especially when aiming to obtain low densities and good foam morphology. In detail, a broad temperature window between the onset of crystallization and gelation is preferred for cooling, and for isothermal processes, an extended time in the gel-like state appeared beneficial.

In conclusion, as editors of this Special Issue, we are very proud of the high-quality papers published and the rigorous review process conducted, providing cutting-edge research results and the latest developments in the fields of polymer science and engineering, including the innovative processing, biopolymers, and characterization of polymer-based products, polymer physics, composites, modeling, simulations and rheology. Therefore, contributions focus on fundamental and experimental results in a thematic range comprising conventional processing technologies as well as innovative processing and material-based macromolecular research. The Special Issue aims to compile the current state-of-the-art and highlight the wide range of applications.
